# The Pkn22 Kinase of *Nostoc* PCC 7120 Is Required for Cell Differentiation via the Phosphorylation of HetR on a Residue Highly Conserved in Genomes of Heterocyst-Forming Cyanobacteria

**DOI:** 10.3389/fmicb.2019.03140

**Published:** 2020-01-21

**Authors:** Baptiste Roumezi, Xiaomei Xu, Véronique Risoul, Yingping Fan, Régine Lebrun, Amel Latifi

**Affiliations:** ^1^Laboratoire de Chimie Bactérienne, CNRS, Aix-Marseille Université, Marseille, France; ^2^Proteomic Platform, Marseille Protéomique IBiSA Labelled, CNRS, IMM, Aix-Marseille Université, Marseille, France

**Keywords:** cell differentiation, cyanobacteria, genomic conservation, Hanks-kinase, phosphorylation

## Abstract

Hanks-type kinases encoding genes are present in most cyanobacterial genomes. Despite their widespread pattern of conservation, little is known so far about their role because their substrates and the conditions triggering their activation are poorly known. Here we report that under diazotrophic conditions, normal heterocyst differentiation and growth of the filamentous cyanobacterium *Nostoc* PCC 7120 require the presence of the Pkn22 kinase, which is induced under combined nitrogen starvation conditions. By analyzing the phenotype of *pkn22* mutant overexpressing genes belonging to the regulatory cascade initiating the development program, an epistatic relationship was found to exist between this kinase and the master regulator of differentiation, HetR. The results obtained using a bacterial two hybrid approach indicated that Pkn22 and HetR interact, and the use of a genetic screen inducing the loss of this interaction showed that residues of HetR which are essential for this interaction to occur are also crucial to HetR activity both *in vitro* and *in vivo*. Mass spectrometry showed that HetR co-produced with the Pkn22 kinase in *Escherichia coli* is phosphorylated on Serine 130 residue. Phosphoablative substitution of this residue impaired the ability of the strain to undergo cell differentiation, while its phosphomimetic substitution increased the number of heterocysts formed. The Serine 130 residue is part of a highly conserved sequence in filamentous cyanobacterial strains differentiating heterocysts. Heterologous complementation assays showed that the presence of this domain is necessary for heterocyst induction. We propose that the phosphorylation of HetR might have been acquired to control heterocyst differentiation.

## Introduction

Protein phosphorylation/dephosphorylation processes play an important role in signal transduction and in regulation of physiological functions in all three domains of life. The nature of the amino-acid phosphorylated depends on the kinase family. The kinases phosphorylating proteins on Serine/Threonine or Tyrosine residues are named Hanks-type kinases (Hanks and Hunter, 1995; Stancik et al., 2018). The phosphorylation of Serine, Threonine, and Tyrosine residues catalyzed by Hanks-type kinases has long been thought to occur in Eukaryotes only. Genome sequencing and physiological studies have shown, however, that this is not the case since genes potentially encoding these kinases have been found to exist in a large number of prokaryotic genomes and to be involved in various cellular processes in several bacterial species (Pereira et al., 2011; Stancik et al., 2018). Recent studies have shown, for example, that the life cycle of *Myxococcus xanthus* is partly controlled by a network of interacting Hanks-type kinases (Munoz-Dorado et al., 1991; Nariya and Inouye, 2006). In *Bacillus subtilis*, spore development and germination are controlled by the YabT and PrkC kinases, respectively (Shah et al., 2008; Bidnenko et al., 2013). The process of morphogenesis is controlled by the StkP kinase in *Streptococcus pneumoniae* (Fleurie et al., 2014). The virulence of several bacteria such as *Mycobacterium tuberculosis* (Cowley et al., 2004), *Mycobacterium pneumoniae* (Schmidl et al., 2010) and *Yersinia pseudotuberculosis* (Galyov et al., 1993) depends on the presence of Hanks-type kinases. A recent phylogenetic analysis has suggested that the prokaryotic and eukaryotic Ser/Thr/Tyr kinases have a common evolutionary origin, which challenges the idea that the prokaryotic proteins may have originated from Eukaryotes (Stancik et al., 2018).

Cyanobacteria, the only Prokaryotes performing oxygenic photosynthesis, form a phylum of diverse bacteria colonizing a wide range of ecological environments. The availability of genome sequences covering the whole phylum (Shih et al., 2013) has made it possible to perform comparative genomic investigations on this group of prokaryotes. A genomic study has shown the presence of Hanks-type kinases encoding genes in 16 of the 21 genomes analyzed. These genes range from 0 to 51 in number, and the largest numbers occur in filamentous diazotrophic strains (Zhang et al., 2007). In an overall study on the phosphoproteome of the unicellular cyanobacterium *Synechocystis* PCC 6803, which possesses seven Hanks-type kinases, 301 phosphorylation events were observed on Ser/Thr/Tyr residues when the bacterium was grown in nitrogen-rich medium, and changes in the global phosphoproteome were found to occur in response to nitrogen starvation (Spat et al., 2015). Protein modifications resulting from Ser/Thr/Tyr phosphorylation may therefore play an important role in the physiology of cyanobacteria. Relatively little is known so far, however, about the signaling pathways in which Hanks-Type kinases and their substrates are involved in cyanobacteria.

The first Hanks-Type kinase to be detected in cyanobacteria was described in the filamentous strain *Anabaena*/*Nostoc* PCC 7120 (referred herein as *Nostoc*) (Zhang, 1993), which possesses a total number of 48 genes potentially coding for kinases of this kind (Zhang et al., 2007). *Nostoc* is a diazotrophic strain which can differentiate a specific cell type responsible for fixing atmospheric nitrogen. When combined nitrogen is abundant *Nostoc* forms long filaments called vegetative cells consisting of a single cell type. When the filaments of *Nostoc* are deprived of combined nitrogen, 5–10% of the vegetative cells differentiate into heterocysts. These micro-oxic cells are semi-regularly distributed along the filaments, which provide a suitable environment for N_2_-fixation. Deprivation of combined nitrogen triggers the accumulation of 2-oxoglutarate (2-OG), the molecular signal inducing heterocyst differentiation (Laurent et al., 2005). Among the various genes involved in the regulatory cascade responsible for heterocyst formation and patterning (Herrero et al., 2016), the global regulator NtcA and the specific master regulator HetR are key transcriptional factors in the cascade resulting in heterocyst development (Herrero et al., 2016). HetR is essential for cell differentiation (Buikema and Haselkorn, 1991). It regulates hundreds of genes in response to combined nitrogen starvation (Mitschke et al., 2011; Flaherty et al., 2014; Videau et al., 2014). HetR exists in different oligomeric states among which dimer and tetramer have been proposed to interact with DNA (Huang et al., 2004; Valladares et al., 2016). The oligomerization of HetR has been shown to be regulated by phosphorylation (Valladares et al., 2016).

Based on genetic studies, the contribution of Hanks-type kinases to the differentiation process at work in *Nostoc* has been described. A mutant strain of the HepS kinase-encoding gene (*all2760*) has been found to show an impairment focusing on the synthesis of the polysaccharide layer surrounding the mature heterocyst (Lechno-Yossef et al., 2006). The alr1336 gene encoding the PknH kinase is required for the connections between heterocysts and vegetative cells, and also for maintaining the heterocyst pattern (Ehira and Ohmori, 2012; Fukushima and Ehira, 2018). Overproduction of the PknE kinase (*alr3732*) inhibits heterocyst development (Saha and Golden, 2011). These findings all suggest that Hanks-type kinases may play a role in several aspects of the *Nostoc* developmental program (Saha and Golden, 2011). However, the activity of these kinases, how they are regulated and the nature of their substrates still remain to be elucidated.

We have previously established that the *pkn22* (*alr2502*) gene encoding a putative Hanks-type kinase is induced by exposure to oxidative stress and to nitrogen starvation in *Nostoc*, and that this kinase connects the cellular responses to these two conditions (Yingping et al., 2015). The transcription of the *pkn22* gene is activated by NtcA when *Nostoc* is deprived of combined nitrogen (Yingping et al., 2015), and the transcription of *hetR* and *ntcA* is not under the control of Pkn22 (Yingping et al., 2015). Here we present genetic evidence that heterocyst differentiation requires the activity of the Pkn22 kinase and that there exist epistatic relationships between Pkn22 and the master regulator HetR. This makes of Pkn22 an important factor involved in regulating the physiology and the metabolism of *Nostoc*.

## Materials and Methods

### Growth Conditions

*Escherichia coli* strains were grown in Luria Broth medium (Euromedex). The plasmids were maintained with Ampicillin (100 μg/ml) or Kanamycin (100 μg/ml).

*Nostoc* sp. PCC 7120 and its derivatives were grown in BG11 medium at 28°C in air under continuous illumination (40 μE m^–2^s^–1^). For growth survey, strains were first cultivated in BG11 medium until OD 750 = 0.5. They were transferred either in BG11 or BG11_0_ after a washing step in the same medium. The *pkn22* insertion mutant and its growth conditions have been described previously (Xu et al., 2003). Cell cultures of *Nostoc* recombinant strains were supplemented with neomycin (50 μg ml^–1^). To avoid overexpression effects of gene transcription from the *petE* promoter, the copper concentration used was 0.4 μM, which is below the concentration triggering a maximal induction level (Buikema and Haselkorn, 2001). Heterocyst formation was induced by transferring the cultures (OD 750 = 0.8) to BG11_0_ (BG11 without sodium nitrate). The growth was maintained for 4 days. The presence of heterocysts was assessed microscopically. The percentage of heterocysts and their spacing in the filaments were rated visually using bright-field microscopy. Heterocysts were distinguished by their thick cell envelope, changes in the granularity of the cytoplasm, and the cyanophycin granules formed at the cell poles. Only the lengths of vegetative cells present between heterocysts along the filaments were recorded as intervals. Over 1,000 total vegetative cells and heterocysts were counted at each time point.

The *Nostoc* strains used in this work are listed in Table 1. The frequency of heterocysts are given in Table 2.

**TABLE 1:** List of the *Nostoc* strains used in this study.

**Strain name**	**Description/Antibiotic resistance**	**Origin**
Wild type	*Nostoc/Anabaena* PCC 7120 wild type strain	Pasteur Cyanobacterial Collection
*pkn22* mutant	*Nostoc* insertion mutant of the *pkn22* gene/(Sp/Sm*^*R*^*)	Xu et al., 2003
*pknC*	*pkn22* mutant containing the pRL-*petE-pkn22* plasmid/(Sp/Sm*^*R*^* and Neo^*R*^)	Yingping et al., 2015
*pkn22/pkn*[K63R]	*pkn22* mutant containing a pRL-*petE-pkn22* plasmid derivative where the *pkn22* gene has been mutated to encode for a K63R substitution/(Sp/Sm*^*R*^* and Neo^*R*^)	This study
*pkn22/hetR*	*pkn22* mutant containing the pRL-*petE-hetR* plasmid/(Sp/Sm*^*R*^* and Neo^*R*^)	This study
*ΔhetR*	*Nostoc* deletion mutant of the *hetR* gene	Borthakur et al., 2005
*ΔhetR/hetR*	*ΔhetR* mutant containing the pRL-*petE-hetR* plasmid/(Neo^*R*^)	This study
*ΔhetR/hetR*[S127A], *ΔhetR/hetR*[S179a], *ΔhetR/hetR*[S130A], *ΔhetR/hetR*[S130D], *ΔhetR/hetR*[P202L], *ΔhetR/hetR*[S127G], *ΔhetR/hetR*[S127G]	*ΔhetR* mutant containing a pRL-*petE* plasmid harboring a *hetR* mutated gene encoding for the amino acid substitutions indicated between brackets/(Neo^*R*^)	This study

**TABLE 2:** Percentage of heterocysts formed by different strains used in this study after combined nitrogen starvation.

**Strain**	**% of heterocysts, 24 h after nitrogen starvation**	**% of heterocysts, 96 h after nitrogen starvation**
Wild type	10–12	10–12
*pkn22* mutant	0	1–2
*pknC*	8–10	10–12
*pkn22/hetR*	12^∗^	10
*ΔhetR*	0	0
*ΔhetR/hetR*	10^∗^	10–12^∗^
*ΔhetR/hetRS130A*	0	0
*ΔhetR/hetRS130D*	22^∗^	22–25^∗^

*The number of the filaments analyzed was 60–100 in average; The conditions were contiguous heterocysts were observed are indicated by an asterisk.*

### Plasmid Construction and Recombinant Protein Purification

The *pkn22* mutant and its derivative strain expressing the *pkn22* gene under the *petE* promoter on the pRL25 plasmid (*pRL-petE-pkn22*, see Table 1) have been previously described (Xu et al., 2003). The catalytic Lysine residue at position 63 of Pkn22 was changed into an Arginine residue using a megaprimer PCR strategy with pRL*pkn22* as the template and the two-step PCR approach using the primers *pkn fw mut*, *pkn rev*, and *pkn22 mut* (Table 3). The resulting fragment was cloned into the *Nde*I and *Eco*RI restriction sites of the *pRLpetE* plasmid to give the pRL-*petE-pkn22*[K63R] (Table 1).

**TABLE 3:** List of the primers used in this study.

**Name**	**Sequence (5′-3′)**	**Experiment**
*pkn rtfw*	AAAACGATTGGTGCAGCGATC	Quantitative RT-PCR analysis
*pkn rtrv*	GCTTATTTTCCGTTCCCGCA	
*rnpB rtfw*	TCGTGAGGATAGTGCCACAG	
*rnpB rtrev*	GGAAGTTTCTTCCCCAGTCC	
*hetR dhfw*	GGATCCCATGAGTAACGACATCGATCTGATC	Two-hybrid assays
*hetR dhrv*	GAATTCTTAATCTTCTTTTCTACCAAACACC	
*pkn dhfw*	GGATCCCATGAGCCTCTGCATAAACCCTCA	
*pkn dhrev*	GAATT CTA CTC TAC ATT GCC GCT ACG CTG	
*hetR ptac fw*	GGATCCGAAGGAGATATACCATGAGTAACGACATCGAT	*pkn22* and *hetR* co-expression in *E. coli*
*hetR ptac rev*	AAGCTTTCAGTGGTGGTGGTG	
*KD pBAD fw*	GAATTCACCATGTGGAGCCACCCGCAGTTCGAAAAAAGCCTCTGCATAAACCCT	
*KD pBAD rev*	AAGCTTCTAGTGCAAATTTTTTTCAAT	
*pkn mut fw*	CAAACAGCTAGAGTCCTCAGAGTCCTAATTAACAATCAT	*pkn22* catalytic residue mutation
*pkn mut rev*	ATGATTGTTAATTAGGACTCTGAGGACTCTAGCTGTTTG	
*hetR pET28 fw*	CATATGAGTAACGACATCGATCTG	*E. coli hetR-his* recombinant strains
*hetR pET28 rev*	GGATCCTTAATCTTCTTTTCTACC	
*hetR S127A fw*	TCGCGCATTCCCGGTACAGCCCTCACAAGTGAAGAAAAA	
*hetR S127A rev*	TTTTTCTTCACTTGTGAGGGCTGTACCGGGAATGCGCGA	
*hetR S179A fw*	GAACATCGCATGGAGTTAGCCGAAGCCCTGGCAGAGCAT	
*hetR S179A rev*	ATGCTCTGCCAGGGCTTCGGCTAACTCCATGCGATGTTC	
*hetR pRL fw*	GGGCCCATGAGTAACGACATCGATCTGATC	*hetR*-phosphoablative substitutions in *Nostoc*
*hetR pRL rev*	GGATCCTTAATCTTCTTTTCTACCAAACACCATTT	
*PhetP forward*	[6FAM]ATTTAGTGGTAAATTCTCTT	EMSA assays
*PhetP reverse*	TGAGTTATACGCTATATCAA

1. In the Two hybrid plasmid construction procedure, the *hetR* (*alr2339*) or *pkn22* (*alr2502*) open reading frames were amplified by performing PCR on *Nostoc* genomic DNA using the primers *hetR dhfw*/*hetR dhrev* and *pkn dhfw*/*pkn dhrev*, respectively. After undergoing a digestion step with *Bam*HI and *Eco*RI, the DNA fragments were cloned into the T18 and T25 plasmids (Euromedex).
2. To co-produce HetR and the catalytic domain of Pkn22 in *E. coli*, the *hetR* ORF was cloned under the previously described *ptac* promoter of the *p33tac* modified plasmid (Bouillet et al., 2017), and the part of the *pkn22* gene encoding the kinase domain (residues 1–325) was cloned under the control of the arabinose promoter of the pBAD24 plasmid. The *hetR* gene was fused to a His-tag sequence. The primers used are specified in Table 3. The two recombinant plasmids were introduced into the TG1 *E. coli* strain. The production of the two recombinant proteins were induced simultaneously using IPTG at 0.5 mM and arabinose at 0.2% overnight at 16°C. The HetR protein was then purified as described previously (Yingping et al., 2015).
3. To express and purify HetR and its variants, the *hetR* ORF was amplified by PCR from *Nostoc* genomic DNA using the primers *hetR pET28 fw/hetR pET28 rev*. After a digestion step with *Nde*I and *Bam*HI, the DNA fragment was cloned into *pET28* plasmid (Novagen), giving *hetR* fused at its N-terminus to a *histidine*-tag. This plasmid was then used as a template to transform the Serine residues at positions 127, 130, and 179 into Alanine using the megaprimer PCR strategy with the primers *hetR S127A fw/hetR S127A rev, hetR S130A fw/hetR S1130A rev*, and *hetR S179A fw/hetR S179A rev*. Likewise, Serine 130 was transformed into Aspartate using the megaprimer PCR strategy with the primers *hetR S130D fw/hetR S1130D rev*. The resulting plasmids were analyzed by performing sequencing procedures, and the plasmid bearing the appropriate sequences were transformed in the BL21DE3 (Novagen) expression strain. The purification of HetR was performed as described previously (Hu et al., 2015).

To produce the phosphoablative HetR variants in *Nostoc*, the *hetR*S127A/D, *hetR*S130A/D and *hetR*S179A/D sequences were amplified from the corresponding *pET28* recombinant plasmids using *hetR pRL fw/hetR pRL rev* as primers. After undergoing digestion with *Apa*I and *Bam*HI, the PCR fragments were cloned into the *pRLpetE* plasmid and conjugated in the *Nostoc ?hetR* strain (Borthakur et al., 2005). The *hetR* genes from *Rivularia* PCC 7116 (Riv7116_3691) and *Oscillatorai nigroviridis* (Osc7112_0139) were synthesized by Eurofins^1^ and cloned into the *pRLpetE* plasmid using the *Bam*HI and *Eco*RI restriction sites. The production of HetR proteins in *Nostoc* was checked by Western blot using anti-HetR antibodies.

### Quantitative RT-PCR

RNA was extracted as previously described (Xu et al., 2003). Chromosomal DNA was removed by treating RNA preparations with 1 μl of DNAse (at 2 U/μl) (Ambion) for 1 h at 37°C. The concentration of RNA was determined spectrophotometrically. Reverse transcription: for each reaction, 1 μl of random hexamer primers (Invitrogen) and 500 ng of total RNA were denaturated at 95°C and chilled quickly on ice. A mix consisting of 4 μl of 5× buffer, 1 μl of RNase Inhibitor (Invitrogen), 1 μl of 5 mM dNTP and 1 μl of MMLV reverse transcriptase enzyme (200 U/μl, Invitrogen) was added in a total volume of 20 μl, followed by 1 h of incubation at 45°C. PCR conditions were identical for all reactions. The 15 μl-reaction mixture consisted of 1× GoTaq qPCR Master Mix (Promega), 0.75 μl of SYBR Green I Dye (Roche), and 500 nM final concentration of each primer. The cDNA resulting from reverse transcription was diluted 25× and used as template. PCR amplifications were carried out in CFX96 qPCR System (BioRad) as described previously (Fan et al., 2014). The primers used in the quantitative-PCR experiments are listed in Table 3. The primers used for analyzing *pkn22* gene expression were chosen downstream to the insertion site of the Spectinomycin resistance cassette used to construct the *pkn22* mutant (Xu et al., 2003). All measurements were carried out in triplicate. The data were analyzed using Software Bio-Rad CFX manager 3.0 (BioRad), and the delta Ct method. Only reactions with over 80% efficiency were considered.

Imidazol was removed from purified proteins using PD10 columns (Healthcare, Orsay, France). Proteins were concentrated on Vivaspin columns and used for subsequent analyses.

### Bacterial Two Hybrid Assays

Bacterial two-hybrid assays were performed as described by Karimova et al. (1998). Briefly, after co-transforming the BTH101 strain with the two plasmids expressing the T18- and T25- fusions, LB plates containing ampicillin and kanamycin were incubated at 30°C for 2 days. 3 ml of LB medium supplemented with ampicillin, kanamycin and 0.5 mM IPTG (Sigma Aldrich) were inoculated and grown at 30°C overnight. ß-Galactosidase activity was determined as previously described (Zubay et al., 1972). The values presented are means of five independent assays on samples containing 10 independent clones each.

### *hetR* Mutagenesis

Random mutagenesis was performed on the *hetR* gene ORF using the GeneMorph II Random Mutagenesis Kit from Stratagene with the primers *hetdhfw*/*hetRdhrev* and *pkn dhfw*/*pkn dhrev*. The PCR fragments were then cloned into the pT25 plasmid (Zubay et al., 1972) using BamH1 and EcoR1 restriction sites. The strain BTH101 previously transformed by pT18-*pkn22* was transformed here by the resulting library of pT25-*hetR* mutants and plated onto LB plates containing Ampicillin and Kanamycin. After being incubated for 2 days at 30°C, colonies were replicated on MacConkey petri dishes and incubated again for 2 days at 30°C. Minipreps of DNA were prepared from the white colonies and used to transform an MC4100 strain to isolate the pT25-*hetR* mutant alone. A further two-hybrid assay was performed between the isolated T25-*hetR* mutant and T18-*pkn22* in the strain BTH101 strain in order to check the loss of the previous interactions. The production of the recombinant T25-HetR mutant proteins was then checked by performing Western blot analysis using antibody directed against HetR. The DNA of clones showing full-length T25-HetR proteins were sequenced and kept for further analysis.

### Sodium Dodecyl Sulfate-Polyacrylamide Gel Electrophoresis (SDS-PAGE) and Immunoblot Analysis

Proteins were fractionated by performing SDS-PAGE (12% except where indicated) stained with Coomassie blue (Euromedex, Souffelweyrshim, France). For electrophoresis under non-reducing conditions, β-mercaptoethanol was omitted from the SDS-PAE gels, and the proteins were not heated before loading into the gel. For immunoblot analysis, the proteins were transferred to nitrocellulose membranes before being revealed with specific polyclonal antibodies. Immune complexes were detected with anti-rabbit peroxidase-conjugated secondary antibodies (Promega) and enhanced chemiluminescence reagents (Pierce, Illkich, France). Anti-HetR antibodies were developed by Covalab and used at a 1:1000 dilution.

### Phosphorylation Assays

HetR (50 μM) or BSA (50 μM) were incubated either under the same experimental conditions used in Valladares et al. (2016) (incubation with 30 μM ATP, 15 μCi [γ-^32^P]-ATP, 25 mM Tris–HCl (pH 7.5), 100 mM KCl, 5 mM MgCl_2_ and 10% glycerol) or submitted to a kinase assay as follows: the proteins were incubated in a phosphorylation buffer (20 mM HEPES (pH 7.2), 10 mM MgCl_2_, 1 mM DTT, 50 μM cold ATP). The reaction was initiated by adding 2 μCi [γ-^32^P]-ATP. As phosphatase inhibitor, PhosStop (Roche) was added to all the phosphorylation assays following the manufacturer indications. The mixtures were incubated during 60 min at 30°C. To examine the ability of HetR to phosphorylate the myelin basic protein (MBP, Sigma), MBP (1 μg) was incubated in the phosphorylation buffer with or without HetR (10 μM). The kinase domain of PrkC of *B. subtilis* (3 μM) was used as a positive control. The reactions were stopped by adding the Laemmli buffer and the proteins were separated by SDP-Page under non-reducing conditions (without heating and without reducing agent in the loading buffer). Radioactive signals from phosphorylated proteins were revealed by autoradiography using a FUJI phosphoimager. The experiment was repeated three times with independent protein purifications and one representative result is shown.

### Electrophoretic Mobility Shift Assays (EMSA)

The promoter region of the *hetP* gene (*alr2818*) was obtained by PCR using *hetP* RT-forward and *hetP* RT-reverse primers (Table 3). The forward primer was modified at its 5’ end by adding the 6-carboxyfluorescein (6-FAM) dye. Purified HetR protein was incubated with the promoter fragments (50 nM) in a buffer containing 10 mM Tris (pH 8), 150 mM potassium chloride, 500 nM EDTA, 0.1% Triton X-100, 12.5% glycerol, 1 mM dithiothreitol and 1 μg DiDC competitor (poly(2’-deoxyinosinic-2’-deoxycytidylic acid) sodium salt), at 4°C for 30 min. The electrophoresis was performed at 250 V for 60 min. The DNA was revealed using Typhoon FLA 9500 (GE Healthcare Life Sciences). The experiment was repeated three times with independent protein purifications and one representative result is shown.

### Mass Spectrometry and Data Analysis

A total of 15 μg of purified HetR were separated by performing SDS-PAGE electrophoresis under non-reducing conditions. Protein-containing bands were subjected to trypsin digestion after several steps: Blue Coomassie stained gel bands were washed with 100 mM acetonitrile/ammonium bicarbonate pH 7.5 for two times of 10 min, and disulfide bond-containing proteins were then reduced by 10 mM dithiothreitol in 100 mM ammonium bicarbonate pH 7.5 for 45 min at 56°C, alkylated by 55 mM iodoacetamide in 100 mM ammonium bicarbonate pH 7.5 in darkness for 20 min at room temperature and digested overnight by a Trypsin/LysC mix (Promega) at 10 ng/μL in 25 mM ammonium bicarbonate pH 7.5 at 37°C. LC-MS/MS analyses were performed on an ESI-Q-Exactive plus mass spectrometer (ThermoFisher) combined with a nano liquid chromatograph (Ultimate3000, Dionex). Tryptic peptide solutions were dried, solubilized again in 8 μL of 0.05% TFA/2% acetonitrile/25%ammonium citrate 100 mM in water (v/v/v) and 6 μL loaded onto a nano trap (Acclaim PepMap100, 100 μm × 2 cm, 5 μm, 100 Å, Dionex) before being eluted onto a C18 column (Acclaim PepMapRSLC, 75 μm × 150 mm, 2 μm, 100 Å, Dionex). A linear gradient from 6 to 40% of mobile phase B (0.1% (v/v) formic acid (FA)/80% (v/v) acetonitrile) in mobile phase A (0.1% (v/v) FA) was applied for 207 min. The peptides were detected in the positive ion mode, using a Top 12 Data Dependent workflow with a 40-s dynamic exclusion. One full scan event MS in the Orbitrap at 70 000, in the 350–1900 m/z range, was followed by a fragmentation MS/MS step at 17 500performed on the top 12 ions in the Higher Energy Collisional Dissociation cell set at 30. Spectra were processed using Proteome Discoverer software (Thermo FisherScientific, version 2.1.0.81) using a workflow including the Sequest HT and MS Amanda algorithms, along with the Percolator node, to validate the Peptide Spectrum Matches (PSM) based on the *q*-Value, and the ptmRS node for optimized phosphosite localization. The search was performed using the sequence of HetR.

### Phylogenetic Analysis

The genomic set analyzed in this study included 160 genomes present in the NCBI database^2^. The complete list of the genomes selected to build the tree is given in Supplementary Table S1. To build the phylogenetic tree of HetR, the genomes cited above were analyzed with BlastP (Altschul et al., 1990) using the sequence form *Nostoc* as a query and an *e*-value < e^–95^. Multiple alignments of the proteins were generated using the Constraint-based multiple Alignment tool (COBALT) (Papadopoulos and Agarwala, 2007). The phylogenetic tree was constructed using the tree generator tool available in the NCBI database. The sequence of *Synechococcus* served to root the tree.

## Results

### The *pkn22* Mutant Was Impaired in Heterocyst Differentiation

**FIGURE 1:**
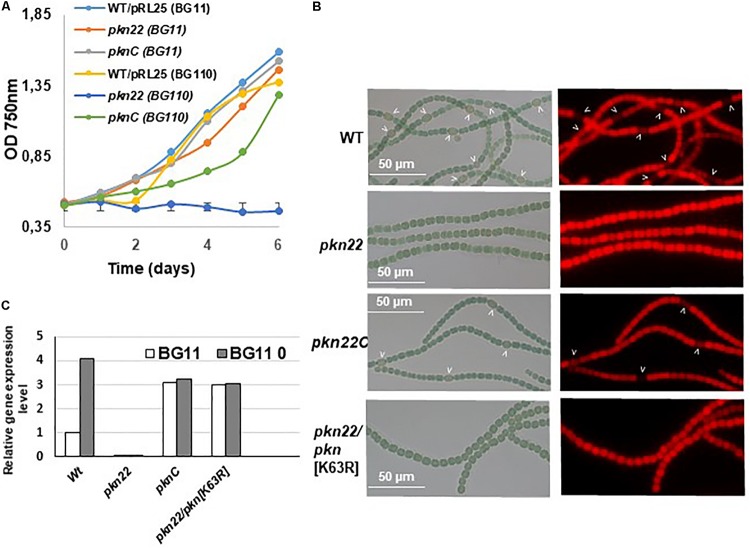
The *pkn22* mutant is unable to sustain diazotrophic growth. **(A)** Growth curve of *Nostoc* strains grown in either BG11 (nitrate-containing medium) or BG11_0_ (nitrate free medium). Each sample was measured in triplicate and error bars give the standard deviations. **(B)** Microscope images of *Nostoc* strains grown for 24 h in BG11_0_. Heterocysts are indicated by arrows. *PknC* stands for the *pkn22* complemented strain and *pkn22/pkn*[K36A] for the *pkn22* strain complemented with the *pkn22* gene bearing the K36A substitution. **(C)** Quantitative RT-PCR analysis of the *pkn22* transcripts in presence (BG11, white bars) or absence of combined nitrogen (BG11_0_, gray bars). Data are expressed as fold-change between normal and starvation conditions. Each sample was measured in triplicate and the standard deviation is indicated by error bars. Values were normalized to the *rnpB* transcript. The value obtained for the Wild type strain in BG11 was set to 1.

**FIGURE 2:**
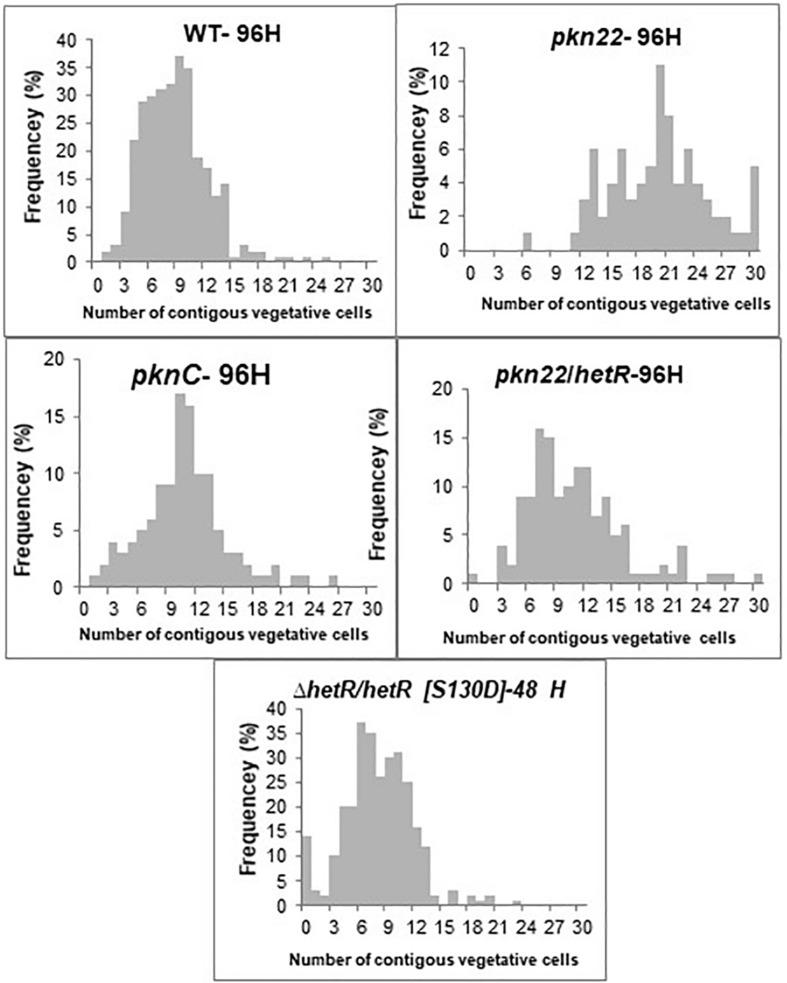
The *pkn22* mutant is not able to develop heterocysts. Heterocyst pattern formation in the wild type, *pkn22* mutant, and *pkn22* derivative strains. Strains were grown in BG11 medium to an OD_750_ of 0.4 and induced to form heterocysts by transfer to BG-110 medium. Vegetative cells and heterocysts were scored microscopically at indicated times after heterocyst induction. The data shown are representative of three independent experiments.

Since the transcription of the *pkn22* gene is induced in response to combined nitrogen starvation and depends on the global transcriptional cellular differentiation activator NtcA (Yingping et al., 2015), we wondered whether this kinase might contribute to the growth under combined nitrogen starvation, and hence to the process of heterocyst development. To answer this question, the growth of the mutant was compared with that of the wild type strain in BG11 medium (with nitrogen) and BG11_0_ medium (without any combined nitrogen). The results presented in Figure 1 show that the growth of the mutant was impaired in the absence of a combined nitrogen source. The introduction of the *pkn22* gene into a replicative plasmid in the mutant (*pknC* strain) (Yingping et al., 2015) partially restored the ability of the mutant to grow in the presence of N_2_ as the sole nitrogen source (Figure 1A). These data indicate that the growth of *Nostoc* requires the presence of the Pkn22 kinase under nitrogen starvation. The heterocyst differentiation process was then analyzed in the *pkn22* mutant in comparison with the wild type strain. Interestingly, the heterocyst formation process was delayed in comparison with what occurred in the wild type strain. No heterocysts were observed during the first 24 h after the nitrogen step-down (Figure 1B and Supplementary Figure S2) in the mutant strain, and a few heterocysts began to appear only 96 h after the step-down (Supplementary Figure S2 and Table 2). These heterocysts did not allow the strain to grow in the absence of combined nitrogen starvation, since the growth of the mutant did not resume after several days in BG11_0_. In order to further examine the *pkn22* mutant phenotype, the pattern of heterocyst occurrence along the filament was analyzed at various times after the nitrogen step-down. The wild type strain showed a mean number of 12 vegetative cells between two heterocysts, 24 h after combined nitrogen starvation, whereas the *pkn22* mutant did not form any heterocysts at all, as mentioned above. Ninety-six hours after the onset of nitrogen starvation, the *pkn22* mutant had formed only a few heterocysts since the average number detected was about 20 vegetative cells between two heterocysts (Figure 2). The *pkn22* gene complemented partially the *pkn22* mutation, since 96 h after the nitrogen step-down, the distribution of heterocysts along the filaments showed a fairly similar pattern to that occurring in the wild type strain, consisting of an average number of 10–12 vegetative cells between two heterocysts (Figure 2). The inability of the *pkn22* mutant to develop heterocysts explains why the mutant was unable to grow in BG11_0_ medium.

### The Catalytic Residue of Pkn22 Contributed Importantly to Cell Differentiation

To investigate how Pkn22 is involved in the differentiation process, we examined how the process of heterocyst development was affected by catalytic residue substitutions. Multiple alignment of the amino acid sequence of Pkn22 with those of other Ser/Thr kinases showed that the Lysine at position 63 corresponds to the conserved residue responsible for ATP binding in this class of kinases (Hanks and Hunter, 1995; Li et al., 1995; Supplementary Figure S1A). The Lysine 63 was substituted to Arginine, the mutated gene was expressed in the *pkn22* mutant and the resulting strain was called *pkn22/pkn*[K63R]. Quantitative RT-PCR analysis were undergone to check that the mutated gene was actually expressed. The data obtained indicated that the *pkn22* transcripts were expressed at similar levels in the PknC and the *pkn22/pkn*[K63R] strain (Figure 1C). Contrary to what was observed with the wild type *pkn22* gene, the ectopic expression of a mutated gene encoding a protein with a K63R substitution did not restore the ability of the *pkn22* mutant to develop heterocysts, or to grow under combined nitrogen starvation conditions (Figure 1B and Supplementary Figure S1B), conceding that the mRNA level of the *pkn*[K63R] reflects the protein level. It was therefore concluded that normal cellular differentiation requires the kinase activity of Pkn22.

### *hetR* Overexpression Compensated for the *pkn22* Mutation

**FIGURE 3:**
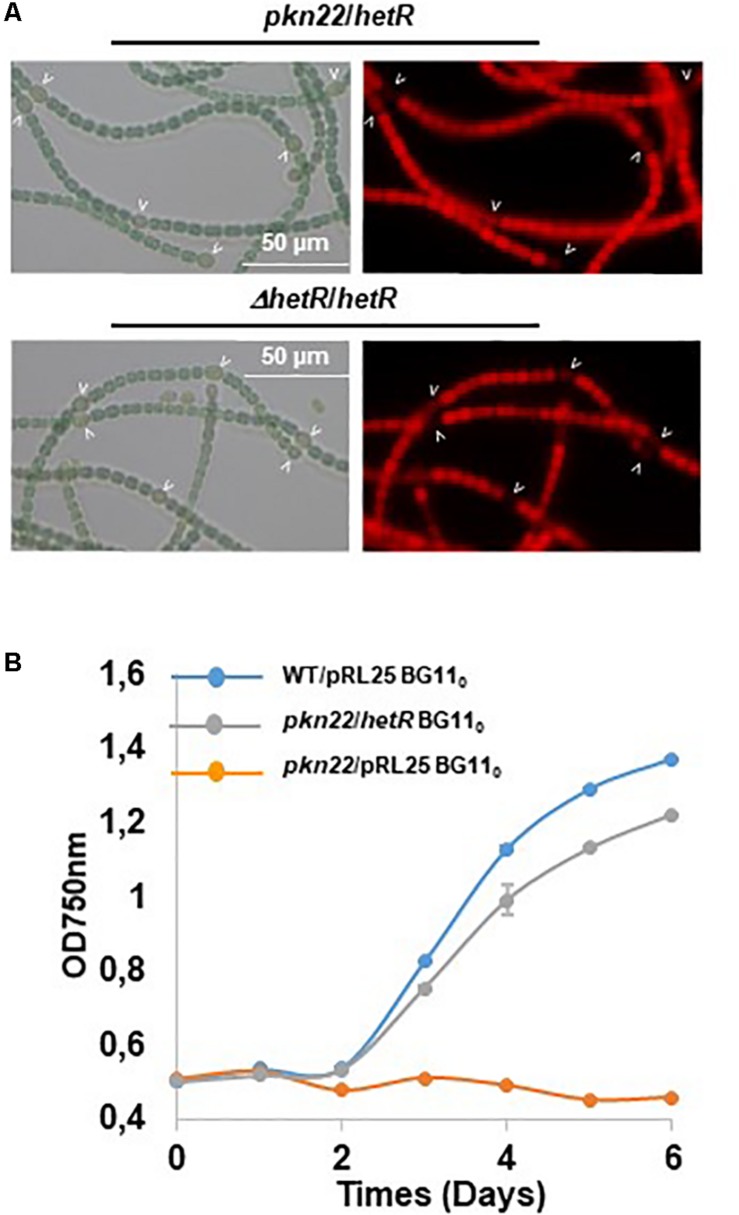
Overexpression of *hetR* rescues the phenotype of the *pkn22* mutant. **(A)** Microscope images of *Nostoc* strains grown in BG11_0_. Heterocysts are indicated by arrows. **(B)** Growth curve of *Nostoc* strains grown for 24 h BG11_0_. Each sample was measured in triplicate and error bars give the standard deviations.

Since the differentiation process was initiated later in the *pkn22* mutant than in the wild type strain, we wondered whether this process might be impaired in the mutant during the initiation of the developmental program. We therefore examined the effects of the ectopic expression of regulatory genes controlling the initiation of heterocyst differentiation in the mutant. The *ntcA* and *hetR* genes encoding the global and specific regulators of heterocyst differentiation, respectively, were expressed in the mutant. The transcription of *ntcA* and *hetR* genes are subjected to autoregulation (Black et al., 1993; Cai and Wolk, 1997; Buikema and Haselkorn, 2001) and are mutually dependent (Muro-Pastor et al., 2002), which might bias the conclusions drawn. We therefore chose to express them from the copper-inducible *petE* promoter that has been used for *hetR* ectopic expression from a replicative plasmid (Buikema and Haselkorn, 2001). Although the overproduction of *ntcA* had no effect (Supplementary Figure S2), the introduction of *hetR* restored the wild-type phenotype, since heterocysts were observed after 24 h and the recombinant strain *pkn22*/*hetR* was able to grow in the absence of combined nitrogen (Figures 3A,B). The *hetR* gene also corrected the defective *pkn22* mutant pattern, since heterocysts had formed in every 10 or 12 vegetative cells in the majority of the filaments 72 h after the nitrogen step-down (Figure 2 and Table 2). The overexpression of *hetR* in the wild type strain has been reported to increase the number of heterocysts along the filaments and to result in contiguous heterocysts (Buikema and Haselkorn, 2001). During the present experiments, we also overexpressed *hetR* in the wild type strain and in the *ΔhetR* strain, and observed the same phenotype as previously described indicating that the *hetR* gene was actually overexpressed under our experimental conditions (Supplementary Figure S2). It was therefore concluded that heterocyst formation requires the presence of the Pkn22 kinase, and that the overexpression of *hetR* can compensate for the absence of this kinase (see section “Discussion”).

### Interactions Between Pkn22 and HetR

**FIGURE 4:**
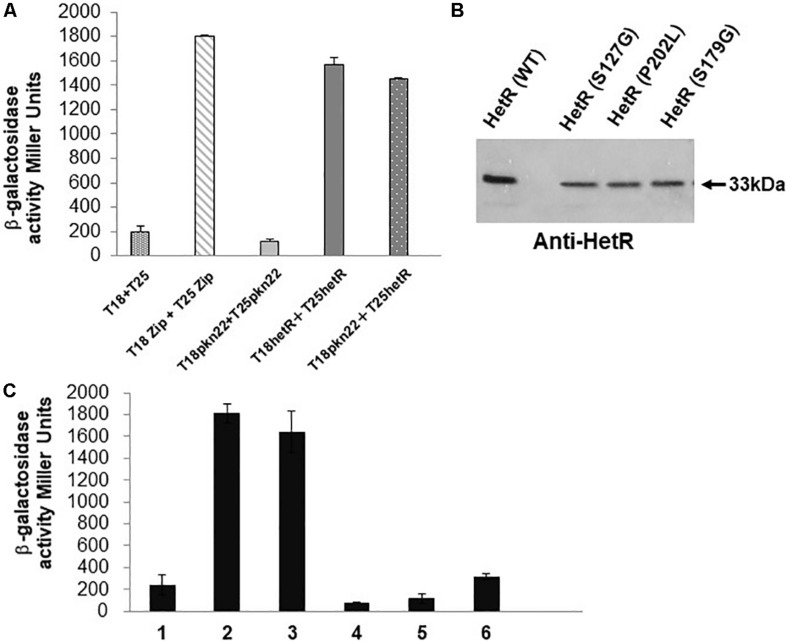
The Pkn22 kinase interacts with HetR. **(A)** Bacterial two hybrid assay between Pkn22 and HetR. BTH101 strain was transformed with pT18*pkn22* and pT25*hetR*, β-galactosidase activities were measured as described in section “Materials and Methods” and were expressed in Miller units. Strains producing the T18 and T25 served as negative control. Strains producing T18Zip and T25 Zip served as positive control. **(B)** Immunoblot analysis of the T25*hetR* variants obtained after random mutagenesis. Cell extracts of BTH101 strains harboring T18-*pkn22* and mutagenized T25-*hetR* were submitted to immunodetection using anti-HetR antibodies. Only full-length peptides were selected for further analysis. **(C)** Bacterial two hybrid assay between T18Pkn22 and the series of T25HetR mutants isolated after random mutagenesis. 1, T18–T25 negative control; 2, T18Zip-T25Zip positive control; 3, HetR-Pkn22; 4, HetR(S127G)-Pkn22; 5, HetR(P202L)-Pkn22; 6, HetR(S179G)-Pkn22.

The existence of a genetic link between Pkn22 and HetR described above suggests that these two proteins may be functionally linked, and it was therefore proposed to determine whether they may interact with each other. For this purpose, bacterial two hybrid assays based on the reconstitution of adenylate cyclase activity were performed in an *E. coli cya* mutant (Karimova et al., 1998). Pkn22 and HetR were fused to the N-terminus of the T18 and T25 domains of *Bordetella pertussis* adenylate cyclase, using the two compatible plasmids pUT18 and pKT25, respectively. In the *cya* strain BTH101, adenylate cyclase activity was restored when the T18-HetR and T25-HetR proteins were produced together (Figure 4A). HetR protein is known to form dimers/tetramers (Kim et al., 2011; Valladares et al., 2016) and the interaction observed between HetR monomers in the bacterial two hybrid assays confirmed the validity of this approach. Pkn22 monomers were not found to interact with each other in our tests (Figure 4A). Adenylate cyclase activity was restored when the T18-Pkn22 and T25-HetR were produced together, and the β-galactosidase activities obtained were almost at the same level as in the positive control sample [the T18 and T25 fused to the leucine zipper region of the yeast protein GCN4 as used in the original system (Karimova et al., 1998), consisting of the proteins from the original system] (Figure 4A). This finding indicates that HetR and Pkn22 interacted with each other in our assays.

**FIGURE 5:**
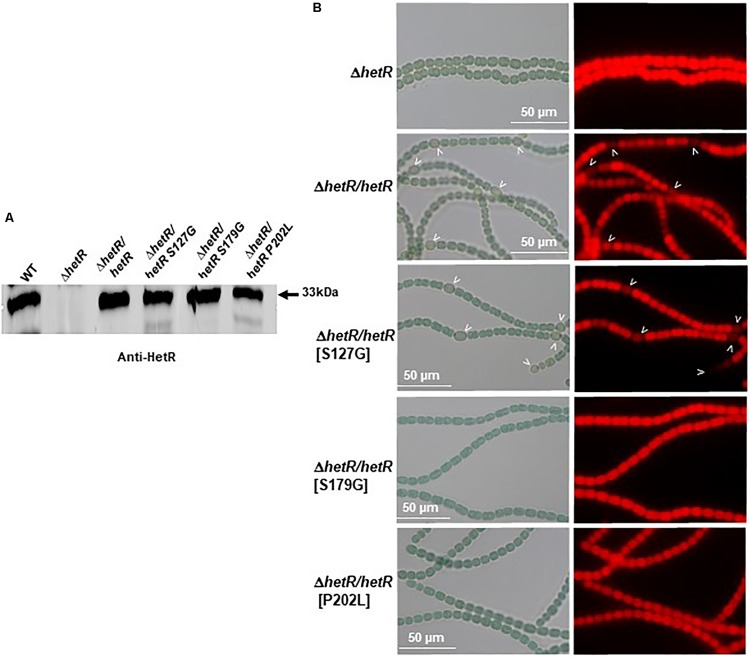
Three residues of HetR involved in the interaction with Pkn22 are also required for normal differentiation. **(A)** Western blot analysis of HetR in *Nostoc* strains. Cells were grown in BG11 medium up to the mid-log growth phase and shifted to BG11_0_ for 24 h. Samples of 75 μg of proteins were used in each assay. The arrow indicates HetR protein. **(B)** Microscope images of *hetR* and *hetR* strain expressing various variants of *hetR*. Strains were grown 48 h in BG11_0_, heterocysts are indicated by arrows.

In order to select HetR variants that could no longer interact with Pkn22, random mutagenesis was performed on the *hetR* coding sequence, which was then cloned into the pT25 plasmid. The resulting library of pT25-*hetR* mutants was then screened using two hybrid method against pT18-*pkn22*, and clones devoid of adenylate cyclase activity were identified. Expression of the recombinant T25-*hetR* mutant genes was then checked by performing Western blot using an antibody directed against HetR. Forty variants of the HetR protein with one or two amino acid substitutions were obtained in all. Clones giving full-length T25-HetR proteins were kept for further analysis (Figure 4B). The plasmids of these clones were extracted and sequenced, and the loss of interactions with Pkn22 was again quantified by performing ß-galactosidase assays (Figure 4C), the mutations harbored by the three clones obtained introduced the following substitutions: S127G, P202L, S179G. With a view to characterizing the physiological consequences of disrupting the Pkn22-HetR interactions, and to avoid the input of *hetR* transcriptional regulation, the *hetR* mutated genes were expressed in a replicative plasmid under the *petE* promoter in a Δ*hetR* strain. The synthesis of these variants in the *hetR* mutant was checked by Western blot analysis (Figure 5A) and the ability of the recombinant strains to develop heterocysts was analyzed (Figure 5B). The expression of the wild type version of *hetR* from the same promoter and the same plasmid was used as a control. The substitutions that affected HetR function and abolished the differentiation process were S179G and P202L. The mutant harboring the S127G substitution was not able to form heterocysts after 24 h, and only a few heterocysts were observed 48 h after the nitrogen step-down (Figure 5B and Table 2). The finding that amino acids involved in the interaction with Pkn22 were also required for HetR to function normally supports the idea that there exists a functional relationship between this Hanks kinase and the master cell differentiation regulator.

### A Phosphorylated Serine Residue Was Crucial to HetR Activity

**FIGURE 6:**
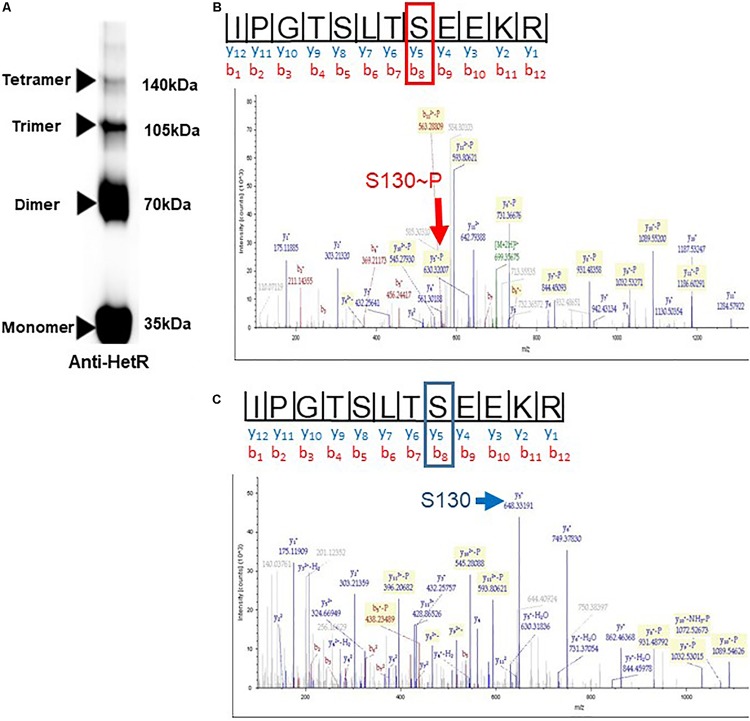
In the presence of Pkn22, HetR is phosphorylated on a Serine residue. **(A)** immunoblot of purified HetR (50 μM) used in the mass spectrometry analysis. The concentration of the gel used was 4–20%. The electrophoresis was undergone on SDS-Page under non-reducing conditions. Several oligomeric forms of HetR were obtained, the monomer, dimer, trimer, and tetramers are indicated by arrows. Same results were obtained when HetR was produced in the absence or presence of Pkn22. **(B,C)** Mass spectrometry analysis of HetR produced in *E. coli* in the presence **(B)** or absence of the kinase domain of Pkn22 **(C)**. After purification, the recombinant HetR protein was subjected to trypsin digestion. The spectra show the fragmentation pattern of the phosphopeptides I_123_PGTSLTSEEKR_134_. The mass increment of the fragment Y4, due to the phosphorylation of Ser 130, is indicated by the red arrow. The blue arrow indicates the fragment Y4 without the mass increment. The data presented in the figure are representative of five independent experiments with the monomer form. Similar data were obtained with the dimeric and trimeric forms. The yield of the tetrameric form obtained was below the concentration needed for mass spectrometry analysis.

**FIGURE 7:**
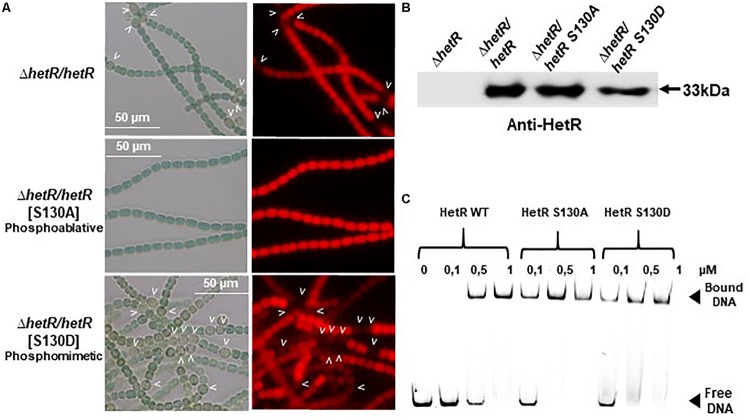
Analysis of phosphoablative and phosphomimetic substitutions of Ser 130 on HetR activity. **(A)** Microscope images of *hetR* and *hetR* strain expressing phosphoablative and phosphomimetic variants of HetRS130. Strains were grown 24 h in BG11_0_, heterocysts are indicated by arrows. **(B)** Western blot analysis of HetR in *Nostoc* strains. Cells were grown in BG11 medium up to the mid-log growth phase and shifted to BG11_0_ for 24 h. Samples of 75 μg of proteins were used in each assay. The arrow indicates HetR protein. **(C)** EMSA analysis of HetR wild type and HetRS130 phosphoablative or phosphomimetic variants. The concentration of the proteins used are indicated. The promoter of *hetP* gene was used as template DNA.

Since Pkn22 belongs to a class of kinases which are potentially able to phosphorylate proteins on Serine or Threonine residues, we wondered whether Pkn22 might phosphorylate HetR, and therefore proposed to test this hypothesis by performing *in vitro* phosphorylation assays. Unfortunately, however, despite a large panel of purification methods tried, we did not succeed in purifying either the full length or kinase catalytic domain of soluble Pkn22. In each assay, the protein was located in inclusion bodies resisting solubilization procedures. Using an alternative approach, we therefore co-expressed the *hetR* and *pkn22* kinase domains in *E. coli* in order to establish whether HetR might be phosphorylated in this background. After production in *E. coli* and purification, HetR was found, as expected, in different oligomeric states (Figure 6A and Supplementary Figure S4). The monomer, dimer and trimeric forms were separately submitted to spectrometry analysis. The results of mass spectrometry analysis showed that when HetR was co-produced with Pkn22, it was phosphorylated on the Ser 130 residue (compare Figures 6B,C). The phosphorylation was detected on the three oligmeric forms of HetR. To characterize the impact of a potential phosphorylation of the Ser 130 residue on HetR activity, phosphoablative (Serine to Alanine) and phosphomimetic (Serine to Aspartate) substitutions were introduced instead of this Ser residue and the mutated genes obtained were expressed in the *hetR* mutant. The impact of these mutations on the differentiation process was deduced from the comparison of the phenotypes of these mutants with that of the *hetR* strain complemented with the wild type version of *hetR* expressed in the same manner than the mutated versions. The data presented in Figure 7A indicate that the HetR S130A phosphoablative variant was not able to complement the *hetR* strain, since no heterocysts were observed. On the other hand, the phosphomimetic variant was found to induce the formation of approximately twofold more heterocysts than the wild type strain (Figures 2, 7A and Table 2) and multiple contiguous heterocysts were observed (Figure 7A). The production of these variants in the *ΔhetR* strain was checked by Western blot using anti-HetR antibodies (Figure 7B). It was therefore concluded that the phosphorylation of the Ser130 residue is crucial to the activity of HetR, and hence to heterocyst development. The ability of HetR to bind to the *hetP* promoter was used to analyze HetR activity *in vitro* (Hu et al., 2015). The phosphoablative and phosphomimetic substitutions performed on Ser 130 did not disrupt the DNA-binding capacity of HetR (Figure 7C), which suggests that the phosphorylation of this residue is unlikely to be a requisite for the interaction with *hetP* promoter to occur.

**FIGURE 8:**
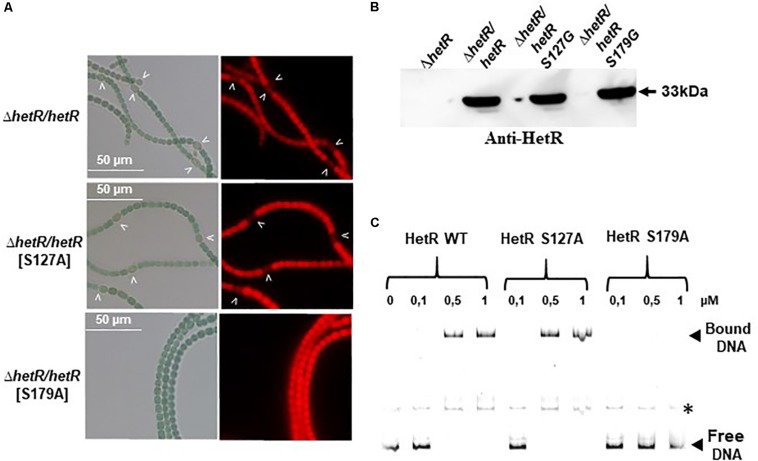
Analysis of phosphoablative substitutions of Ser 127 and Ser 179 on HetR activity. **(A)** Microscope images of *ΔhetR* and *ΔhetR* strain expressing phosphoablative the HetRS127 and HetRS179 variants. Strains were grown 24 h in BG11_0_, heterocysts are indicated by arrows. **(B)** Western blot analysis of HetR in *Nostoc* strains. Cells were grown in BG11 medium up to the mid-log growth phase and shifted to BG11_0_ for 24 h. Samples of 75 μg of proteins were used in each assay. The arrow indicates HetR protein. **(C)** EMSA analysis of HetR wild type, HetRS127 and HetRS179 phosphoablative variants. The concentration of the proteins used are indicated. The promoter of *hetP* gene was used as template DNA. Note that *hetP* promoter (bound or free) presented a secondary band, indicated by an asterisk, which might correspond to a different secondary structuration of the DNA.

Since the Ser 127 and Ser 179 residues were identified in the genetic screen for the loss of HetR ability to interact with Pkn22, the impact of their phosphoablative substitutions was analyzed. Their synthesis in the *ΔhetR* strain was checked by Western blot using anti-HetR antibodies (Figure 8B). The S179A substitutions abolished the capacity of HetR to induce heterocyst formation (Figure 8A) and to bind to *hetP* promoter *in vitro* (Figure 8C). To establish whether this residue is actually phosphorylated *in vivo*, or whether the effect observed was due to the similarity between this residue and the DNA binding motif, further investigations are required. In the case of Ser 127, the phosphoablative substitution performed here did not affect the DNA binding activity of HetR or its ability to induce heterocyst differentiation (Figures 8A,C), which suggests that this residue is probably not phosphorylated *in vivo.*

### Conservation of Serine 130 Residue in Cyanobacteria Forming Heterocysts

**FIGURE 9:**
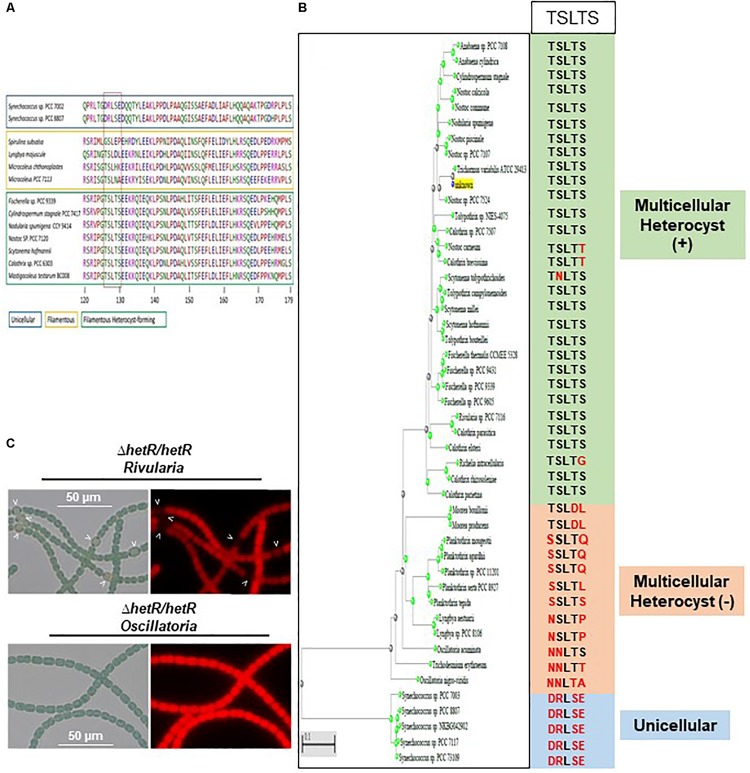
The Serine 130 is part of a sequence highly conserved in strains able to differentiate heterocysts. **(A)** Amino acid Alignment of HetR sequence surrounding the [TSLTS] sequence. The strains able to differentiate heterocysts are framed in green. Filamentous strains not forming heterocysts are framed in yellow. Unicellular strains are framed in blue. **(B)** Phylogenetic tree of HetR showing the conservation of the [TSLTS] sequence in the cyanobacterial genomes analyzed. **(C)** Heterologous complementation assays of the *ΔhetR Nostoc* strain. Strains were grown 24 h in BG11_0_, heterocysts are indicated by arrows.

Sequence alignment and phylogenetic analysis of the HetR sequence across the cyanobacterial phylum showed that Ser 130 belongs to a sequence [TSLTS] which is highly conserved in the cyanobacterial strains which are able to differentiate heterocysts (*Nostocales* and *Stigonematales*) (Figures 9A,B and Supplementary Figure S3). The phosphorylation of HetR might therefore occur in diazotrophic cyanobacteria other than *Nostoc* PCC 7120. The possibility that the presence of the [TSLTS] sequence would be important for HetR to induce heterocyst differentiation was analyzed. For this purpose, the *hetR* sequences from the multicellular non-heterocyst forming cyanobacterium *Oscillatoria nigroviridis*, which does not have the [TSLTS] motif, and the one from the filamentous heterocyst-forming cyanobacterium *Rivularia* PCC 7116, which possesses this sequence (Figure 9B and Supplementary Figure S3) were expressed in the *hetR* mutant of *Nostoc*. Results of Figure 9C show that while *hetR* gene from *Rivularia* complemented the *hetR* strain since heterocysts were observed 24 h after nitrogen stepdown, *hetR* from *Oscillatoria* was not able to complement this mutant (Figure 9C). Apart from the [TSLTS] motif, the sequences of three proteins share high level of conservation with HetR from *Nostoc* displaying 72.57% identity with its homolog in *Rivularia* and 75.25% with HetR from *Oscillatoria* (Supplementary Figure S3). It was therefore concluded that the [TSLTS] sequence in HetR protein might indeed be important for heterocyst development.

## Discussion

**FIGURE 10:**
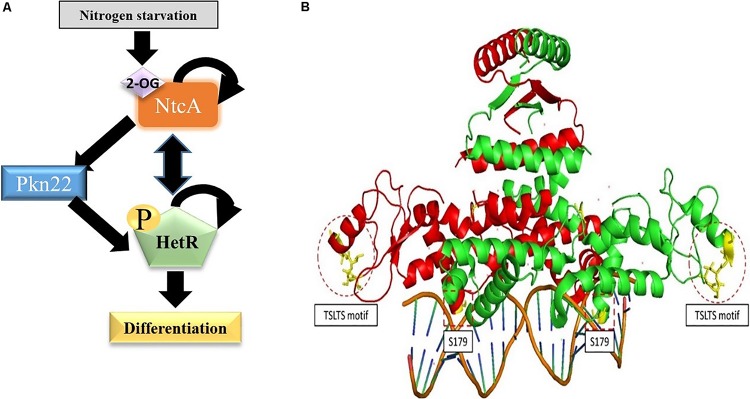
Position of Ser residues 130 and 179 in the structure of HetR. **(A)** The initiation cascade involved in heterocyst differentiation including the Pkn22 kinase. **(B)** The Structure of HetR dimer (PDB number: 4YRV) is adapted from Hu et al. (2015). The two Monomers are respectively colored in green and red. For each monomer, the Serine residues studied in this work are colored in yellow and surrounded by dotted lines. The copyright permission for the adaptation of this figure was obtained from the authors.

The data presented in this paper indicate that the Pkn22 kinase is required for normal heterocyst development to occur via the phosphorylation of the master regulator HetR. In line with this conclusion, an insertion mutant of the *pkn22* gene was unable to form heterocysts within 24 h, as well as being unable to grow under N_2_-regime. We have previously established that the transcription of the *pkn22* gene is under the control of the couple NtcA-2OG. It is therefore likely that the activity of this kinase might occur during the early steps of the developmental program, making it a new player in the initiation cascade that triggers heterocyst formation (Figure 10A).

The genetic and biochemical data obtained in this study brought to light the existence of a connection between Pkn22 and the master regulator of heterocyst differentiation: HetR. Pkn22 seems to be epistatic to HetR, since the overexpression of the *hetR* gene was able to compensate for the absence of Pkn22. In bacterial two component systems, where signal transduction is mediated by the phosphorylation of the response regulator protein by the sensor kinase, the overproduction of the response regulator compensates for the kinase activity of the sensor (for a recent review on the subject, see Jacob-Dubuisson et al., 2018). A similar mechanism may explain why increasing the amount of HetR triggered cell differentiation in the *pkn22* mutant background. In this context, the ability of the two proteins to interact and the fact that residues present in HetR which are required for the interaction with the kinase are also required for the differentiation process point to the existence of physical and functional relationships between them. The fact that two Serine residues (S127 and S179) were found to be required for HetR and Pkn22 interaction raised the question whether HetR might be phosphorylated on Serine residues. The phosphoablative substitution of S179 was found to inhibit the heterocyst differentiation process. If the residue S179 was actually phosphorylated *in vivo*, it would be interesting to establish whether Pkn22 is responsible for this phosphorylation event or whether it is mediated by another kinase. Three other S/T kinases have been reported to be involved in heterocyst differentiation (Lechno-Yossef et al., 2006; Saha and Golden, 2011; Ehira and Ohmori, 2012). It would be worth determining whether any crosstalk among them and Pkn22 might contribute to the functional role of HetR.

HetR has been shown to be phosphorylated when incubated with radioactive adenosine triphosphate (ATP) (Valladares et al., 2016). Consequently, before analyzing the possible phosphorylation of HetR by Pkn22, we wondered whether HetR would be able to act itself as a kinase by analyzing its ability to catalyze phosphotransfer *in vitro*. The data presented in Supplementary Material, where the PrkC kinase from *B. subtilis* was used as a positive control (Shah et al., 2008), indicated that HetR does not possess a kinase activity similar to that of Ser/Thr/Tyr kinases (Supplementary Figure S3B) and can consequently be used as a substrate in a phosphorylation test *in vitro* to analyze its putative phosphorylation by Pkn22. HetR was found to be phosphorylated on the S130 residue only when it is co-produced with Pkn22 in *E. coli* (Figure 6B). Phosphoablative substitution of S130 inhibited the process of heterocyst formation, while strains harboring phosphomimetic substitution of this residue formed larger numbers of heterocyst compared to the strain expressing the wild type version of HetR (Figures 2, 7A and Table 2). This finding suggests that the phosphorylation of this residue is required for HetR to function *in vivo*. The fact that the phosphoablative substitution of S130 did not abolish the DNA binding activity (Figure 7C) suggests that the phosphorylation of this serine may be necessary for interactions to occur between HetR and the RNA polymerase or other protein partners. Indeed, this residue is located in the Flap domain, which is exposed in the structure and has been thought to be required for protein-protein interactions to be possible (Kim et al., 2011; Hu et al., 2015; Figure 10B). On the other hand, S179 is located near the DNA binding motif (Figure 10), which may explain the impact of a post-translational modification of this residue on the interaction of the protein with the promoter. We attempted to purify HetR from *Nostoc* at various times during the differentiation process with a view to analyzing its phosphorylation state, but the amount of protein obtained was below that required for mass spectrometry purposes. It is conceivable that the whole population of HetR proteins does not have to be phosphorylated to initiate differentiation, which would also be a limiting factor for the mass spectrometry analysis of HetR purified from *Nostoc*. It is planned to use other approaches such as phosphoproteome analysis in the future in order to study more closely how HetR phosphorylation contributes to the differentiation process.

Previous RNA seq and ChIp Seq analyses have shown that the transcription of genes expressed in the heterocysts and vegetative cells requires the presence of HetR (Mitschke et al., 2011; Flaherty et al., 2014). It is tempting to imagine that a post-translational HetR modification might constitute one of the mechanisms responsible for the cell-type specificity of this regulator. In this respect, it has been reported that shortly after the onset of nitrogen starvation, HetR protein shows a higher isoelectric point than under combined-nitrogen conditions (Zhou et al., 1998); phosphorylation may be involved in this post-translational modification in response to combined nitrogen starvation. In line with this idea, HetR has been found phosphorylated when incubated with radioactive ATP *in vitro* (Valladares et al., 2016). Moreover, this phosphorylation has been reported to inhibit the accumulation *in vitro* of the tetrameric form which has been postulated to be the active regulatory form in the heterocyst (Valladares et al., 2016). This *in vitro* phosphorylation has been suggested to be either catalyzed by HetR or by a kinase form *E. coli* which could be co-purified with HetR (Valladares et al., 2016). The regulation of heterocyst differentiation is a spatio-temporal regulated process during which a dynamic of HetR phosphorylation could be proposed to occur based on Valladares et al. (2016), study and the data presented in our work: in response to combined nitrogen starvation, NtcA activation by 2-OG, induces *pkn22* expression which leads to HetR phosphorylation and initiation of the developmental program (Figure 10B). When the heterocyst reaches maturity, the stimulation of the autophosphorylation activity of HetR, or the activation of another kinase, limits its regulatory action. Analyzing the phosphorylation/dephosphorylation of HetR separately in the vegetative cells and in the heterocysts through the developmental program will give more insight in the mechanism of this master regulator.

Interestingly, the residue S130 that was found to be phosphorylated in *E. coli* only when HetR was co-produced with Pkn22 belongs to a motif [TSLTS] which is conspicuously highly conserved in the *Nostocales* and *Stigonematales* strains (Figure 9). The phosphorylation of HetR described in the present study might therefore also occur in diazotrophic cyanobacteria other than *Nostoc* PCC 7120. In addition, the genomes of strains belonging to the *Nostocales* and *Stigonematales* species contain at least one copy of the Hanks-type kinase gene (Zhang et al., 2007). The acquisition of the [TSTLS] motif by HetR sequences and its phosphorylation might therefore be an evolutionary step toward the occurrence of heterocyst differentiation and diazotrophy. The fact that HetR from *Rivularia*, which harbors the [TSLTS] sequence, complemented the *ΔhetR Nostoc* mutant, while that from *Oscillatoria* strain which does not contain this sequence did not (Figure 9) is in agreement with this hypothesis. Further studies on the phosphorylation state of HetR in heterocyst-forming strains other than *Nostoc* in comparison with unicellular strains will yield deeper insights into the functional role of HetR and its speciation in the course of evolution.

HetR can therefore be added to the hitherto rather short list of transcriptional regulators (other than two-component systems) that are phosphorylated by Hanks-type kinases. The other known examples on this list are the global gene regulator AbrB (Kobir et al., 2014) and the fatty-acid-displaced regulator FatR (Derouiche et al., 2013) in *B. subtilis*, and the anti-sigma RseA of *M. tuberculosis* (Barik et al., 2010). The interplay between response regulators and Hanks-type kinases is definitely a topic worth investigating, since studies on these lines will shed further light on how the phosphorylation process serves in bacteria to detect and transduce environmental signals.

## Data Availability Statement

All datasets generated for this study are included in the article/Supplementary Material.

## Author Contributions

AL conceived and designed the study and wrote the manuscript. BR, XX, VR, and YF performed the research. AL and RL supervised the research. AL, BR, and RL analyzed the data.

## Conflict of Interest

The authors declare that the research was conducted in the absence of any commercial or financial relationships that could be construed as a potential conflict of interest.

## Footnotes

1 https://www.eurofins.fr/

2 https://blast.ncbi.nlm.nih.gov/Blast.cgi
