# Supplementary material for: The Pkn22 Kinase of Nostoc PCC 7120 Is Required for Cell Differentiation via the Phosphorylation of HetR on a Residue Highly Conserved in Genomes of Heterocyst-Forming Cyanobacteria
Source: Front Microbiol. 2020 Jan 21;10:3140. doi: 10.3389/fmicb.2019.03140 (PMC6985446; doi:10.3389/fmicb.2019.03140)
Supplement: Supplementary file 1 [file Data_Sheet_1.PDF]

SUPPLEMENTAL DATA

Supporting Table 1:

List of the cyanobacterial genomes analyzed in this study and the accession number of their HetR sequences in the NCBI database. The conservation/variation of the TSLTS sequence is indicated in the column entitled “Motif”

Supporting information:

To test the ability of HetR to act as a kinase, HetR protein was incubated in the same experimental conditions than used by Valladares et al (22) or in a kinase assay where the phosphotransferase activity of the kinase is measured in a buffer containing higher concentrations of cold ATP and a low concentration of radioactive ATP (49). The serum albumin bovine (BSA), which does not possess any phosphorylation site was used as a negative control (FigureS3 A, B). While HetR was found in a phosphorylate state when incubated with radioactive ATP (Figure S3B line 1), which is consistent with the data of Valladares et al., it was not able to catalyze phototransfer in the kinase activity assay described above since it was found, like BSA, non-radioactive (Figure S3 B, line2). The phosphotransferase activity of HetR was assessed by analyzing its capacity to phosphorylate the Myelin basic protein (MBP) (50). In this assay, the kinase domain of PrkC of *Bacillus subtilis* was used as a positive control. The phosphorylation of MBP was observed only when it was incubated with PrkC (Figure S3 C). It was therefore concluded that HetR does not possess a kinase activity similar to that of Ser/Thr/Tyr kinases and can consequently be used as a substrate in a phosphorylation test *in vitro* to analyze its putative phosphorylation by Pkn22

Supporting legends to figures

Figure S1

A: Alignment of the amino acid sequence kinase domain of Pkn22 with other Ser/Thr kinases (PkA: *Mus musculus*, PrkC: *Bacillus subtilis*, PknB: *Mycrobacterium tuberculosis*). The conserved amino acids involved in binding of ATP are highlighted in yellow. The conserved amino acids involved in substrate binding and catalysis are in red and blue. The conserved Lysine that was substituted to Arginine is indicated by the arrow.

B: Growth curve of *Nostoc* strains grown in BG110. *PknC* stands for the *pkn22* complemented strain and *pkn22/*

pkn[K63R] for the *pkn22* strain complemented with the *pkn22* gene encoding for the K36A substitution.

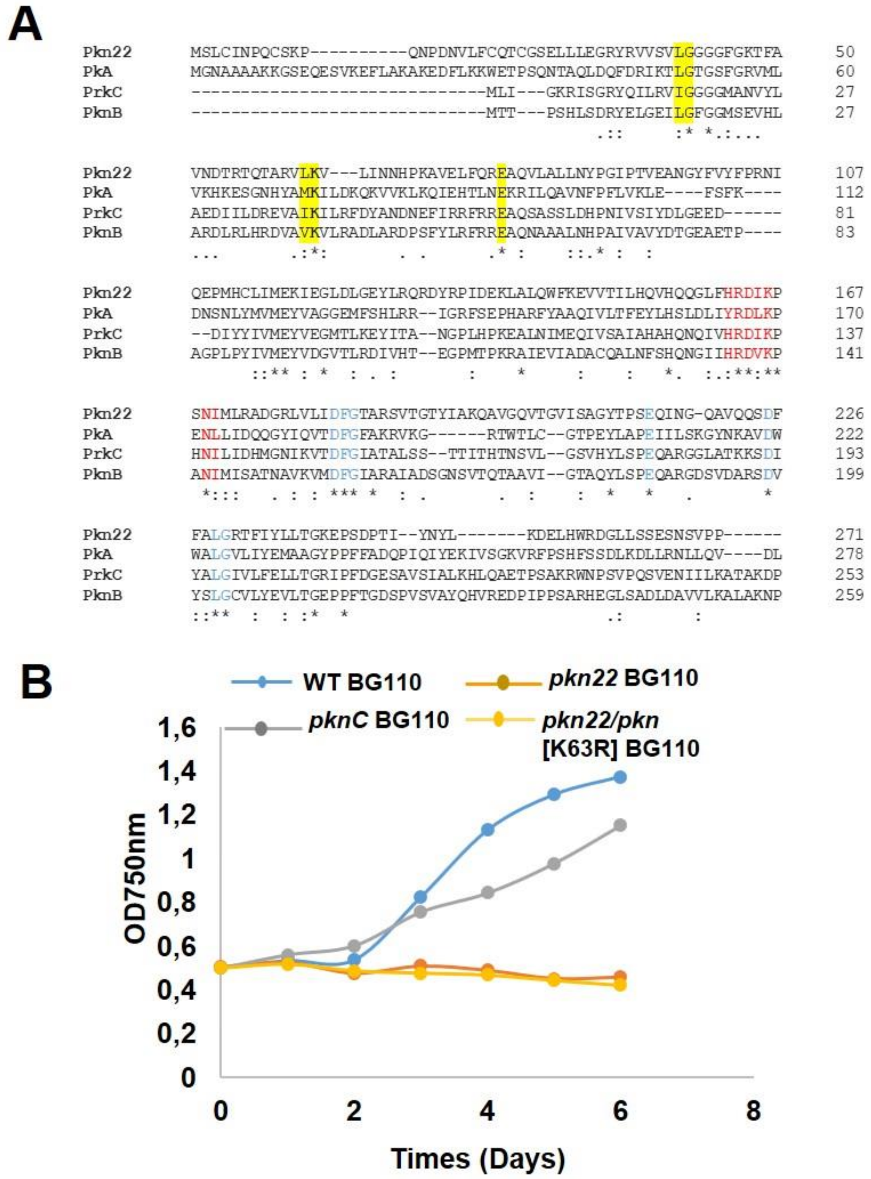

Figure S2

Microscope images of the *pkn22* mutant at different times after nitrogen starvation, the *pkn22/pRL petE-ntcA* strain after 48h growth in $BG11_0$, and the wild type strain bearing the pRL*petE-hetR* after 24h growth in $BG11_0$

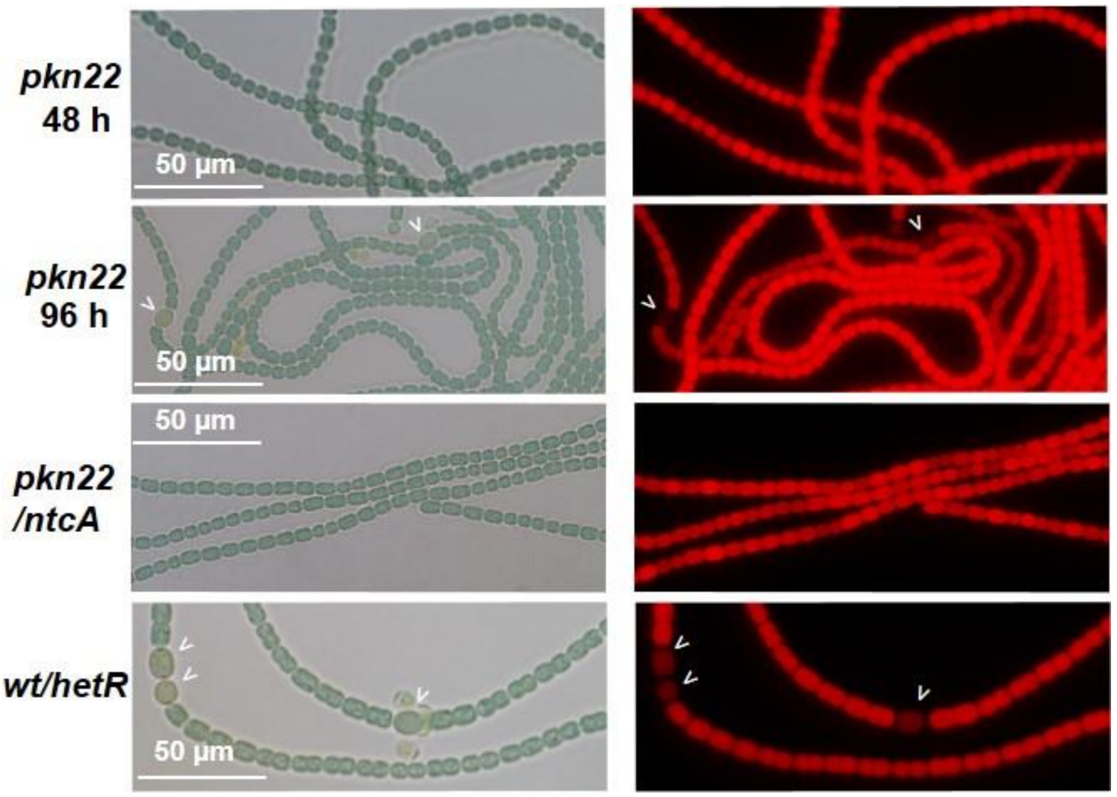

Figure S3
A: Alignment of the amino acid sequence of HetR from *Nostoc*, *Rivularia* PCC7116 and *Oscillatoria nigro-viridis.* The TSLTS sequence is indicated in red. The accession numbers of the proteins are given in Supp Table 1.

B-D HetR is not able to catalyze phosphotransfer**:** Analysis of phosphorylation of HetR
B: HetR and BSA (66 kDa) SDS-Page electrophoresis revealed by Coomassie blue: **1**: HetR, assay[a]; **2**: BSA, assay[a]; **3**: HetR, assay[b]; **4**: BSA, assay[b]

C: HetR and BSA SDS Page electrophoresis revealed by autoradiography: **1**: HetR, assay[a]; **2**: HetR, assay[b], **3**: BSA, assay[a]; **4**: BSA, assay[b]

Assay[a]: the proteins were incubated with incubation with 30µM ATP, 10µCi [γ-^{32}P]-ATP

Assay[b]: the proteins were incubated with incubation with 50µM cold ATP, and 2µCi [γ-^{32}P]-ATP (see text for the details)

D: Analysis of phosphorylation of MBP

MBP (18,5 KdA) was incubated with HetR or with the kinase domain of PrkC (40 kDa). The SDS-Page gel (20%) was revealed by autoradiography

1: MBP, 2 MBP incubated with PrkC, MBP incubated with HetR

A

```
Rivularia       ------------MHNDTDLIKRLDPSAMDQIMLYLAFSAMRTSGHRHGAFLDAAATAAKC
Nostoc          ------------MSNDIDLIKRLGPSAMDQIMLYLAFSAMRTSGHRHGAFLDAAATAAKC
Oscillatoria    MPPNLAISGIVMKNDSLDLVKSLSPSAMDQIMLYLAFSAMRTSGHRHGAFLDAAATAAKC
                              :. **:* *.************************************

Rivularia       AIYMTYLEQGQNLRMTGHLHHLEPKRVKAIVEEVRQALTEGRLLKMLGSQEPRYLIQFPY
Nostoc          AIYMTYLEQGQNLRMTGHLHHLEPKRVKIIVEEVRQALMEGKLLKTLGSQEPRYLIQFPY
Oscillatoria    AIYMTYIEEKNNLRMTGHLHHIEPKRVKVIVEEVQEALTQGKLLKMLGSQEPRYLIQFPY
                ******:*: :**********:****** *****::** :*:*** **************

Rivularia       VWMEKYPWQPGRSRIPGTSLTSEEKRQIEQKLPPNLPDAQLITSFEFLELIEFLHKRSQE
Nostoc          VWMEQYPWIPGRSRIPGTSLTSEEKRQIEHKLPSNLPDAQLVTSFEFLELIEFLHKRSQE
Oscillatoria    VWLEQYPWLPGRPRIPGNNLTADEKKYLEGKIPPNPPDAQLINSFQFMELIEFLHRRSQE
                **:*:*** ***.****..**::**: :* *:*.* *****:.**:*:*******:****

Rivularia       DLPARHQMPLSEALAEHIKRRLLYSSTVTRVDSPWGMPFYALTRPYYATASDEERTYIMV
Nostoc          DLPPEHRMELSEALAEHIKRRLLYSGTVTRIDSPWGMPFYALTRPFYAPADDQERTYIMV
Oscillatoria    DMSPERRMPLSEALAEHIKRRLIYSGTVTRIDAPWGMPFYALTRATYSPAEQEERTFTMV
                *:...::* *************:**.****:*:***********. *:.*.::***: **

Rivularia       EDTARFFRMMREWSERQRHTMRVLEEMDIPPERIDEALEDLDQIIRAWADKYHEVGGAPM
Nostoc          EDTARYFRMMKDWAEKRPNAMRALEELDVPPERWDEAMQELDEIIRTWADKYHQVGGIPM
Oscillatoria    EDTARYFRLMKDWAEKQPKVMRIMETLDIPPERLDQALEELDEIIRNWADRYHKRGEPTM
                *****:**:*::*:*:: :.** :* :*:**** *:*:::**:*** ***:**: *  .*

Rivularia       ALQMVFGKKED-
Nostoc          ILQMVFGRKED-
Oscillatoria    ILQMVFGPQDDA
                 ****** ::*
```

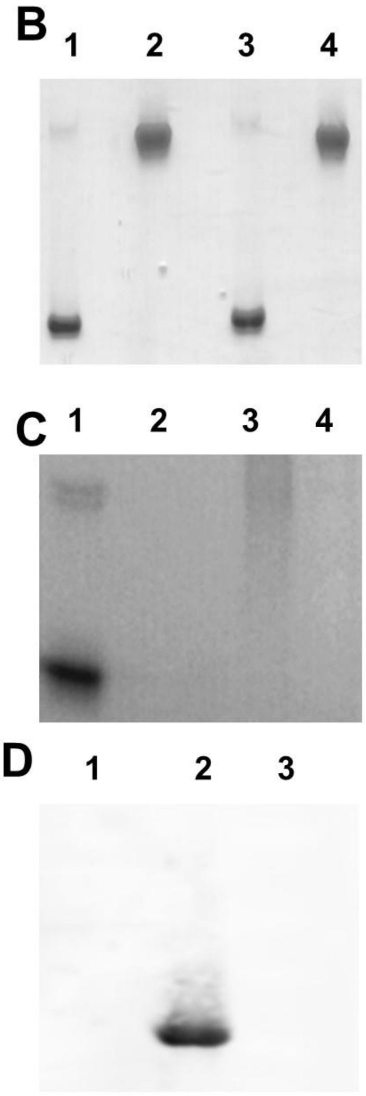

Figure S4

Western blot analysis of HetR protein purified from *Escherichia coli*

500 ng of proteins were loaded on a 4-20% SDS PAGE gel. The electrophoresis was run under non-reducing conditions (the samples were not heated and the loading buffer did not contain β-mercaptoethanol)

1: HetR purified from the BL21DE3 strain (- alkaline phosphatase)

2: HetR purified from the BL21DE3 strain (+ alkaline phosphatase)

3: HetR purified from the TG1 strain, non-producing the Pkn22 kinase domain (- alkaline phosphatase)

4: HetR purified from the TG1 strain, producing the Pkn22 kinase domain (+ alkaline phosphatase)

5: HetR purified from the TG1 strain, producing the Pkn22 kinase domain (- alkaline phosphatase)

6: HetR purified from the TG1 strain, producing the Pkn22 kinase domain (+ alkaline phosphatase)

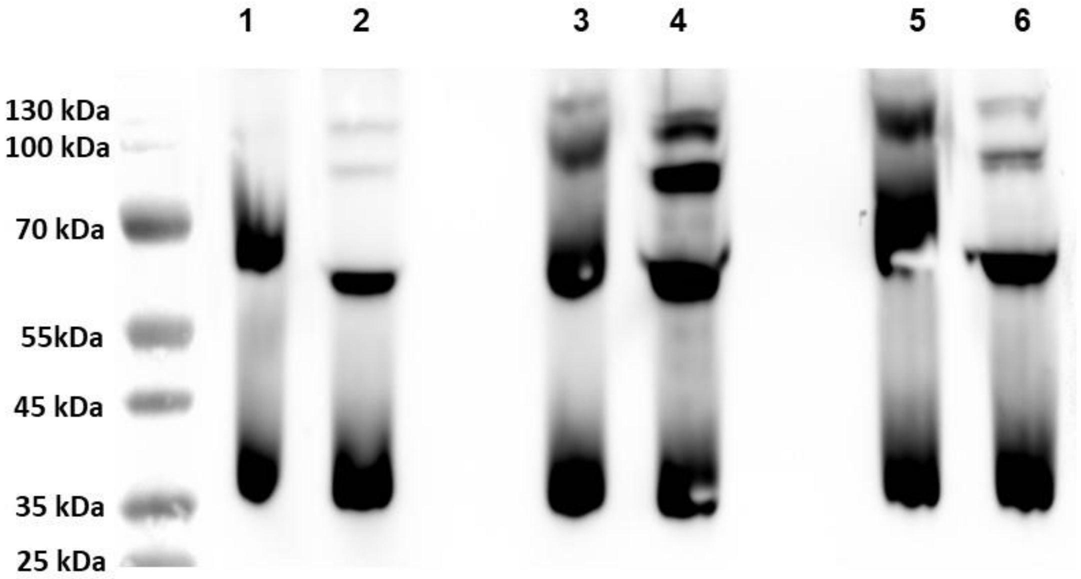

Figure S5

Full length gels for figures 4B, 5A, 6A

Figure S6

Full length gels for figures 7B, 7C

Figure S7

Full length gels for figures 8B, 8C

Figure S4

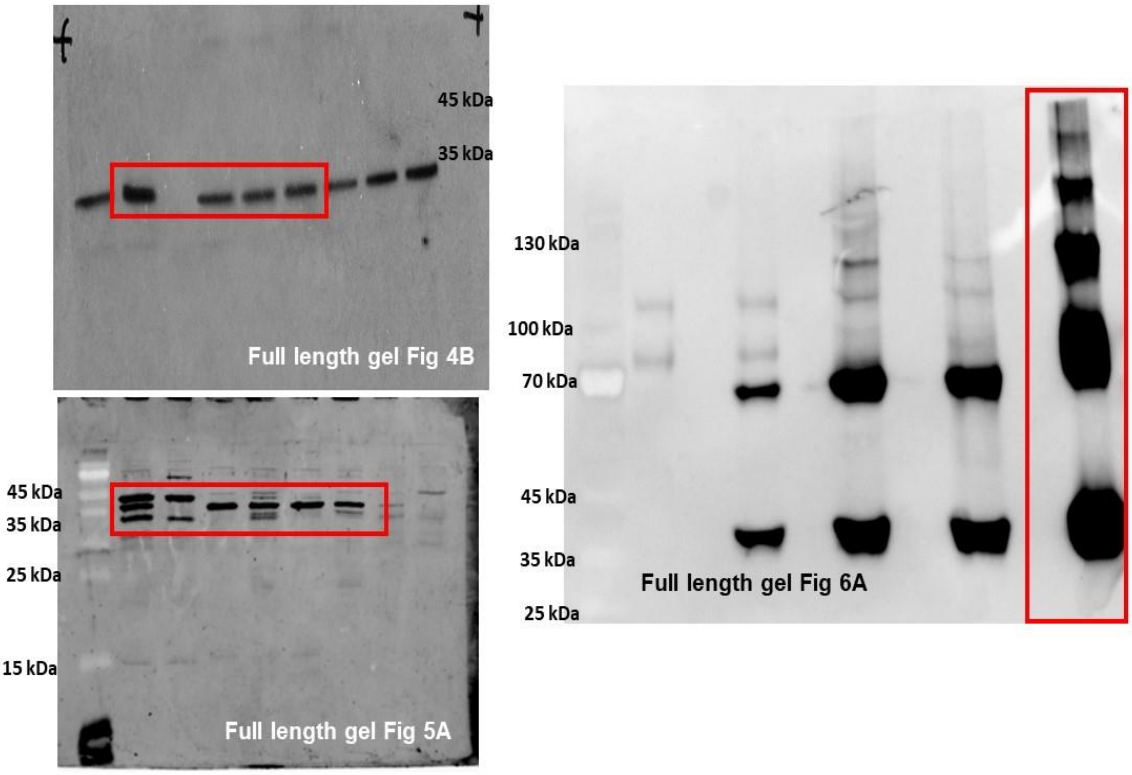
45 kDa
35 kDa
Full length gel Fig 4B
45 kDa
35 kDa
25 kDa
15 kDa
Full length gel Fig 5A
130 kDa
100 kDa
70 kDa
45 kDa
35 kDa
25 kDa
Full length gel Fig 6A

Figure S5

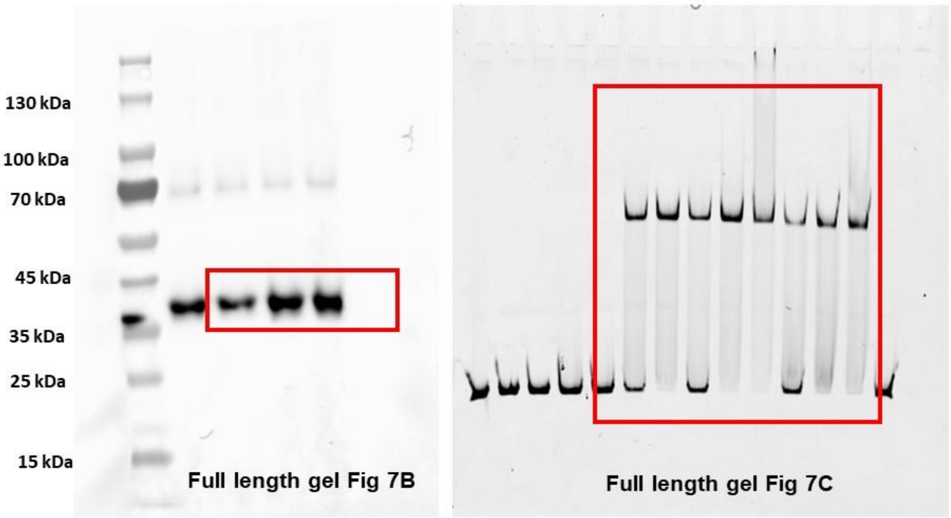
130 kDa
100 kDa
70 kDa
45 kDa
35 kDa
25 kDa
15 kDa
Full length gel Fig 7B
Full length gel Fig 7C

Figure S6

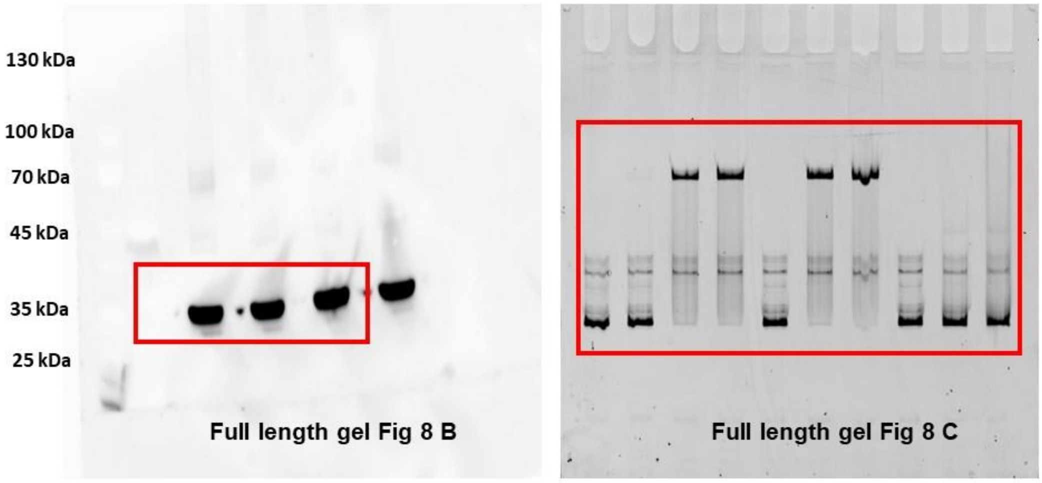
130 kDa
100 kDa
70 kDa
45 kDa
35 kDa
25 kDa
Full length gel Fig 8 B
Full length gel Fig 8 C
